# Longitudinal Evaluation of Changes in Retinal Architecture Using Optical Coherence Tomography in Achromatopsia

**DOI:** 10.1167/iovs.63.9.6

**Published:** 2022-08-05

**Authors:** Magdalini Triantafylla, Eleni Papageorgiou, Mervyn G. Thomas, Rebecca McLean, Susanne Kohl, Viral Sheth, Zhanhan Tu, Frank A. Proudlock, Irene Gottlob

**Affiliations:** 1 Ulverscroft Eye Unit, Neuroscience, Psychology and Behaviour, Robert Kilpatrick Clinical Sciences Building, Leicester Royal Infirmary, University of Leicester, United Kingdom; 2 Molecular Genetics Laboratory, Institute for Ophthalmic Research, Department for Ophthalmology, University of Tübingen, Tübingen, Germany; 3 Department of Neurology, Cooper University Hospital, Cooper Neurological Institute, Cooper Medical School of Rowan University, Camden, New Jersey, United States

**Keywords:** achromatopsia, CNGA3, CNGB3, hyporeflective zone, optical coherence tomography (OCT)

## Abstract

**Purpose:**

This prospective study investigates longitudinal changes in retinal structure in patients with achromatopsia (ACHM) using optical coherence tomography (OCT).

**Methods:**

Seventeen patients (five adults, 12 children) with genetically confirmed *CNGA3*- or *CNGB3*-associated ACHM underwent ocular examination and OCT over a follow-up period of between 2 and 9.33 years (mean = 5.7 years). Foveal tomograms were qualitatively graded and were segmented for quantitative analysis: central macular thickness (CMt), outer nuclear layer thickness (ONLt), and size of the foveal hyporeflective zone (vertical HRZ thickness: HRZt and horizontal HRZ width: HRZw) were measured. Data were analyzed using linear mixed regression models. Both age and visit were included into the models, to explore the possibility that the rate of disease progression depends on patient age.

**Results:**

Fifteen of 17 patients (88%) showed longitudinal changes in retinal structure over the follow-up period. The most common patterns of progression was development of ellipsoid zone (EZ) disruption and HRZ. There was a significant increase in HRZt (*P* = 0.01) and HRZw (*P* = 0.001) between visits and no significant change in CMt and ONLt. Retinal parameters showed no difference in changes by genetic mutation (*CNGA3* (n = 11), *CNGB3* (n = 6)).

**Conclusions:**

This study demonstrates clear longitudinal changes in foveal structure mainly in children, but also in adults with ACHM, over a long follow-up period. The longitudinal foveal changes suggest that treatment at an earlier age should be favored.

Achromatopsia (ACHM), or rod monochromatism is a rare autosomal recessive disorder with an incidence of approximately one in 30,000.^1^^,^^2^ It is characterized by decreased visual acuity, photophobia, nystagmus, impaired color discrimination, absent or severely reduced cone function, and usually normal-appearing fundus.^1^^,^^2^ ACHM is genetically heterogenous, and to date causative mutations have been identified in the *CNGA3*, *CNGB3*, *GNAT2*, *PDE6C*, *PDE6H,* and *ATF6* genes.^3^^–^^7^ Considerable phenotypic variation in functional and imaging findings has been observed within the two most common ACHM genotypes, *CNGA3* and *CNGB3*.^8^^–^^11^

Optical coherence tomography (OCT) has demonstrated variable macular structures in patients with ACHM, including a normal appearance, ellipsoid zone (EZ) disruption, which often presents as a hyporeflective zone (HRZ), a hyperreflective zone that can precede EZ disruption, outer retinal atrophy with retinal pigment epithelium (RPE) loss, and foveal hypoplasia.^10^^–^^16^ The recent advances in the ability to image the human retina from infancy have raised controversy with regard to the stationary nature of ACHM. Several studies are now suggesting it is a progressive condition, which is also supported by corresponding animal models.^13^^,^^17^^,^^18^ The first clinical evidence originated from retrospective studies reporting a deterioration in cone function, visual acuity, and macular changes from infancy to adulthood.^8^ Thiadens et al.^12^ proposed that cone cell loss began in early childhood, with retinal thinning showing a strong association with age thereafter. Thomas et al.^14^ also found that the presence of a HRZ and outer nuclear layer (ONL) thinning were age dependent. These findings were later addressed via longitudinal studies, confirming that dynamic retinal changes occur, especially in younger patients.^13^^,^^18^ On the other hand, Genead et al.^9^ suggested that cone loss is not age dependent, and Sundaram et al.^10^ found that the variable OCT appearances, total foveal retinal thickness and ONL thickness were also not age dependent. Subsequent longitudinal studies concluded that OCT retinal parameters in ACHM either remained stable or changed minimally in a small proportion of patients.^15^^,^^16^^,^^19^^,^^20^

The first human gene replacement trials in ACHM have shown a good safety profile and cone photoreceptor activation in adult patients.^21^ Hence, it is of utmost importance to examine longitudinal structural changes to define the potential window for therapeutic intervention, identify eligible patients, and assess therapeutic efficacy.^7^ An age-dependent retinal thinning in ACHM would underline the importance of early treatment to preserve or restore visual function, and four ongoing clinical gene replacement trials are designed to recruit pediatric patients as well (NCT02599922, NCT02935517, NCT03278873, NCT03758404). The aim of this prospective, observational study is to investigate longitudinal changes of retinal architecture, by means of OCT, in patients with molecularly proven ACHM, over a long follow-up period.^13^^,^^15^^,^^16^

## Material and Methods

### Patients

Seventeen patients (five male and 12 female) with genetically confirmed ACHM, and at least two years’ follow-up with OCT were included in the study (Table). Mean age at first visit was 17 years (range, three months to 63 years). Mean age at the final examination was 20.7 years (range, 3.8–65.7 years). There were five adults (range, 23–63 years) and 12 children (range, three months to 10 years). Mean follow-up period was 5.7 months (range, 2–9.33 months).

**Table.** Summary of Patient Characteristics and Clinical Findings

ID/Sex	Age at Baseline	FU (mo)	Gene	Allele 1/Allele 2	Foveal Hypoplasia	OCT Grade^*^ Baseline	OCT Grade^*^ Last Visit	Change in HRZ Width (Degrees) OD/OS	Change/Year in HRZ Width (Degrees) OD/OS	Change in HRZ Thickness (µm) OD/OS	Change/Year in HRZ Thickness (µm) OD/OS	Change in Foveal ONL Thickness (µm) OD/OS
1/F^†^	45y	108	*CNGA3*	c.1279C>T;p.Arg427Cys/c.1706G>A;p.Arg569His	Y	1	2	+1.5°/+1.8°	+0.2°/+0.2°	+14/+29	+1.6/+3.2	+4/−3
2/F^†^	42y 2m	112	*CNGA3*	c.1279C>T;p.Arg427Cys/c.1706G>A;p.Arg569His	Y	4	5	Excluded	Excluded	Excluded	Excluded	Excluded
3/M	8y 11m	102	*CNGA3*	c.1641C>A;p.Phe547Leu/c.1641C>A;p.Phe547Leu	N	1	2	+1.8°/+1.9°	+0.2°/+0.2°	+22/+24	+2.5/+2.8	−11/−4
4/F	63y 1m	31	*CNGB3*	c.1148del;p.Thr383Ilefs*13/c.257del;p.Pro86Leufs*39	Y	4	4	+0.01°/+0.01°	+0.004°/+0.004°	−6/0	−2.3/0	−2/−6
5/M	4y 7m	90	*CNGA3*	c.848G>A;p.Arg283Gln/c.848G>A;p.Arg283Gln	Y	1	2	+2.5°/+2.1°	+0.3°/+0.4°	+19/+19	+2.6/+3.7	−36/−11
6/F	7y 1m	68	*CNGB3*	c.1148del;p.Thr383Ilefs*13/c.1148del;p.Thr383Ilefs*13	N	1	2	+1.3°/+1.9°	+0.2°/+0.3°	+14/+14	+2.5/+2.5	+7/+21
7/F	1y 11m	57	*CNGA3*	c.1641C>A;p.Phe547Leu/c.1641C>A;p.Phe547Leu	N	3	4	+0.3°/+0.2°	+0.06°/+0.06°	+31/+29	+6.6/+8.2	−7/−5
8/M	8y 6m	67	*CNGA3*	c.1279C>T;p.Arg427Cys/c.107_110del;p.His36Argfs*136	N	1	1	0°/0°	0°/0°	0/0	0/0	−2/−5
9/F	22y 7m	93	*CNGA3*	c.1641C>A;p.Phe547Leu/c.1641C>A;p.Phe547Leu	Y	2	4	+1.3°/+0.7°	+0.2°/+0.1°	+9/+31	+1.2/+4.0	+11/−1
10/M	10y	101	*CNGA3*	c.340G>T;p.Glu114*/c.1641C>A.p.Phe547Leu	Y	2	3	+2.5°/+2.5°	+0.3°/+0.3°	−3/−4	−0.4/−0.5	−18/+2
11/M	9y 9m	69	*CNGA3*	c.567-11G>A:p.?/c.567-11G>A;p.?	N	1	2	+1.7/+1.8	+0.3°/+0.3°	+24/+26	+4.2/+4.5	−6/−12
12/F	3m	42	*CNGB3*	c.886_896del11insT;p.Thr296Tyrfs*9/c.1397T>A;p.Met466Lys	N	3	4	+1.1°/+0.8°	+0.3°/+0.2°	+26/+26	+7.5/+7.5	+38/+24
13/F	45y 1m	80	*CNGB3*	c.1148del;p.Thr383Ilefs*13/c.1148del;p.Thr383Ilefs*13	N	4	5	+7.7°/+3.3°	+1.2°/+0.5°	+41/+20	+6.0/+3.0	+4/+3
14/F	2y	24	*CNGA3*	c.848G>A;p.Arg283Gln/c.1116del;p.Val373*	N	2	4	+0.2°/+0.6°	+0.1°/+0.3°	+2/+5	+1.2/+2.4	−2/−7
15/F	2y 2m	31	*CNGB3*	c.1148del;p.Thr383Ilefs*13/c.1148del;p.Thr383Ilefs*13	Y	2	4	mi/+0.07°	mi/+0.03°	mi/0	mi/0	mi/0
16/F	9y 2m	24	*CNGB3*	c.1148del;p.Thr383Ilefs*13/c.1426C>T;p.Gln476*	Y	3	4	+0.3°/mi	+0.2°/mi	0/mi	0/mi	−10/
17/F	7y 1m	43	*CNGA3*	c.848G>A;p.Arg283Gln/c.848G>A;p.Arg283Gln	N	2	3	+1.6°/+3.5°	+0.5°/+1.0°	0/+2	0/+0.7	−7/0

FU, follow-up; bin, binocular; y, years; m, months; Y, yes; N, no; HRZ, hyporeflective zone; mi, missing value.

Variant designation based on NCBI Reference Sequence NM_001298.2 for *CNGA3* and NM_019098.4 for *CNGB3*.

* OCT grading system according to Sundaram et al.^10^

† Siblings.

The study adhered to the tenets of the Declaration of Helsinki and was approved by the Leicestershire, Northamptonshire and Rutland Committee for the National Research Ethics Service, UK, as well as the Ethics Board of the Medical Faculty, Eberhard Karls University Tübingen under the study no. 116/2015BO2. All patients or legal representatives provided informed consent.

### Clinical Assessment

All patients underwent ophthalmologic examination, including best-corrected visual acuity (BCVA), slit-lamp examination, color vision assessment, dilated fundoscopy and spectral-domain OCT (SD-OCT) imaging on each visit. Electrophysiology was performed according to the International Society for Clinical Electrophysiology of Vision Standards. The diagnosis of ACHM was based on clinical criteria and was confirmed by molecular genetic testing. The clinical characteristics of patients at first visit are shown in the Table.

### OCT Acquisition

Patients underwent spectral OCT imaging acquired with Copernicus (SOCT Copernicus; OPTOPOL Technology S.A., Zawiercie, Poland) or hand-held OCT in young children (HH-OCT, LEICA Envisu system, Leica microsystems, Wetzlar, Germany) according to protocols described in Supplementary 1.^14^^,^^18^^,^^22^^–^^26^ OCT scans were obtained binocularly at each visit following pupillary dilation. Good reliability of measurements between the two devices has been previously reported.^23^

### Qualitative Analysis of Foveal Morphology

Foveal structure on OCT images was graded into 1 of 5 categories using the classification described by Sundaram et al.:^10^ (1) continuous EZ, (2) EZ disruption, (3) EZ absence, (4) presence of an HRZ, (5) outer retinal atrophy, including RPE loss (to use international nomenclature in our publication, the term “inner segment ellipsoid (ISe)” used by Sundaram et al.,^10^ was replaced by EZ) [Staurenghi]. Presence of foveal hypoplasia was defined as the continuation of one or more inner retinal layers (outer plexiform layer, inner nuclear layer, inner plexiform layer, or ganglion cell layer) at the fovea. For each patient both right and left eyes were graded at first and final visit by two independent masked examiners (TM and TZ). OCT images were randomized before analyses. Interexaminer reliability was described with intraclass correlation coefficients. Consensus grading between the two examiners was reached via discussion.

### Quantitative Analysis of Retinal Layers on OCT

Acquired images were exported from the OCT software and imported into ImageJ software (available at rsbweb.nih.gov/ij/).^27^ Retinal layer borders were positioned manually at the fovea by locating points that were fitted with a spline fit. The fovea was identified by visual inspection of the scans for its characteristic features; the deepest point of the foveal pit, thinning of the inner retinal layers, widening of the ONL and lengthening of the photoreceptor outer segments. The central foveal scan was selected for analysis in order to define horizontal dimensions of the foveal HRZ in degrees (HRZ width, HRZw), and vertical dimensions of the foveal HRZ in µm (HRZ thickness, HRZt). In addition, central macular thickness in µm (CMt) and foveal ONL thickness in µm (ONLt) were also measured by manually identifying retinal layers at the center of the fovea, using the protocol described by Mohammad et al.^28^ ONLt was measured between the deepest point of the foveal pit and the external limiting membrane. CMt was the sum of ONLt, the inner segment and the outer segment layer and was measured between the deepest point of the foveal pit and the RPE. All parameters were measured at first and final visit. If the patient had EZ disruption without a HRZ according to the previous classification scheme,^10^ the dimensions of EZ disruption were also calculated as HRZw (in degrees) and HRZt (in micrometers). This approach was used as the site of EZ disruption can be considered as a small HRZ and measuring the size of this EZ disruption enables a more detailed quantitative assessment.^26^ A conversion factor provided by Leica Microsystems (Leica, Wetzlar, Germany) was used to convert lateral (i.e., horizontal) distances to visual angles (288 µm/degree in adult eyes). In a recent study characterizing the time course of development of the optic nerve head region in the early years of life using HH-OCT, we found that parameters such as optic disc and cup width remain constant with age when expressed as a visual angle rather than as a lateral distance measurement.^25^ We suggest this is because these parameters increase proportionally with increasing axial length and that the expansion of the scleral shell is an important factor determining growth of the cup and disc in the first two years of life.^25^

### Molecular Genetic Testing

All patients underwent genetic research testing. Blood samples were sent to the Institute for Ophthalmic Research in Tübingen for genetic investigation in a research setup. Whole genomic DNA was extracted using standard procedures. Genetic analysis of *CNGB3* and *CNGA3* was performed as described previously.^3^^–^^5^ Only patient ID11 carrying the homozygous putative novel splice site variant c.567-11G>A was sequenced for all known ACHM genes (*CNGA3*, *CNGB3*, *GNAT2*, *PDE6C*, *PDE6H* and *ATF6*).

### Statistical Methods

Linear mixed regression models were applied at SPSS software version 24.0 (SPSS, Chicago, IL, USA), to calculate the longitudinal changes in CMt (micrometers), ONLt (micrometers), HRZt (micrometers), and HRZw (degrees), including age, visit (first or second), eye (left or right), and gene mutation (*CNGB3* or *CNGA3*). An interaction term between age and visit was also included in the model, in order to distinguish changes due to age-related retinal development from changes due to disease progression. All analyses were considered significant at a type 1 probability value of *P* < 0.05.

## Results

### Molecular Genetics

Eleven patients (65%) had biallelic mutations in the *CNGA3* gene (three adults, eight children), and six patients (35%) in the *CNGB3* gene (two adults, four children) (Table). Most mutations are already published, yet two novel variants were observed. The *CNGA3* variant c.340G>T;p.Glu114* represents a nonsense mutation truncating the CNGA3 polypeptide well before all structural and functional domains; yet more likely the transcript will be degraded by the mechanism of nonsense-mediated decay. The putative splice site variant c.567-11G>A in intron 5 was observed homozygously in patient ID11 and is predicted to result in a weakening of the canonical splice site and a generation of a cryptic splice site, potentially resulting into the exonification of nine additional nucleotides into exon 6. The effect of gene mutation on retinal parameters was not significant (data not shown).

### BCVA and Foveal Hypoplasia

Mean binocular BCVA at first visit was 0.79 logMAR, and was not significantly different from mean binocular BCVA at the final visit, 0.82 logMAR (paired *t*-test; *P* = 0.33). Age was not correlated with BCVA (Spearman's correlation coefficient = −0.063, *P* = 0.81). Atypical foveal hypoplasia was found bilaterally in eight of 17 (47%) patients. BCVA was similar between patients with or without foveal hypoplasia (paired *t*-test; *P* = 0.19). No correlation was found between BCVA and any of the studied OCT parameters (data not shown).

### Central Macular Thickness and Foveal ONL

Mean CMt was 169 ± 34.3 µm (range, 88.8–232 µm) at first visit and 161.9 ± 26.6 µm (range, 110.4–209 µm) at the final visit (Fig. 2, Fig. 3A). The change in CMt between visits was not significant (*P* = 0.279) (mean change per year = −0.97 µm), although the change in CMt with age approached statistical significance (*P =* 0.056).

**Figure 1.**
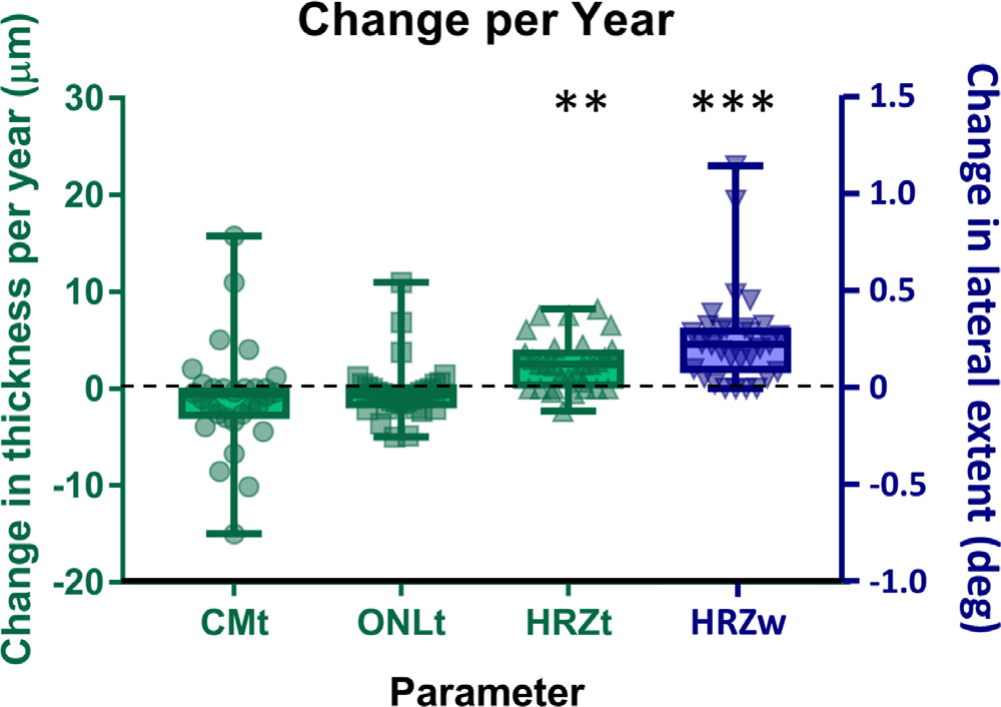
Box-and-whisker plot showing the change/year for CMt, ONLt, HRZt and HRZw. The changes in CMt and ONLt were not significant. There was a significant increase in HRZt and HRZw. **P = 0.01, ***P = 0.001.

**Figure 2.**
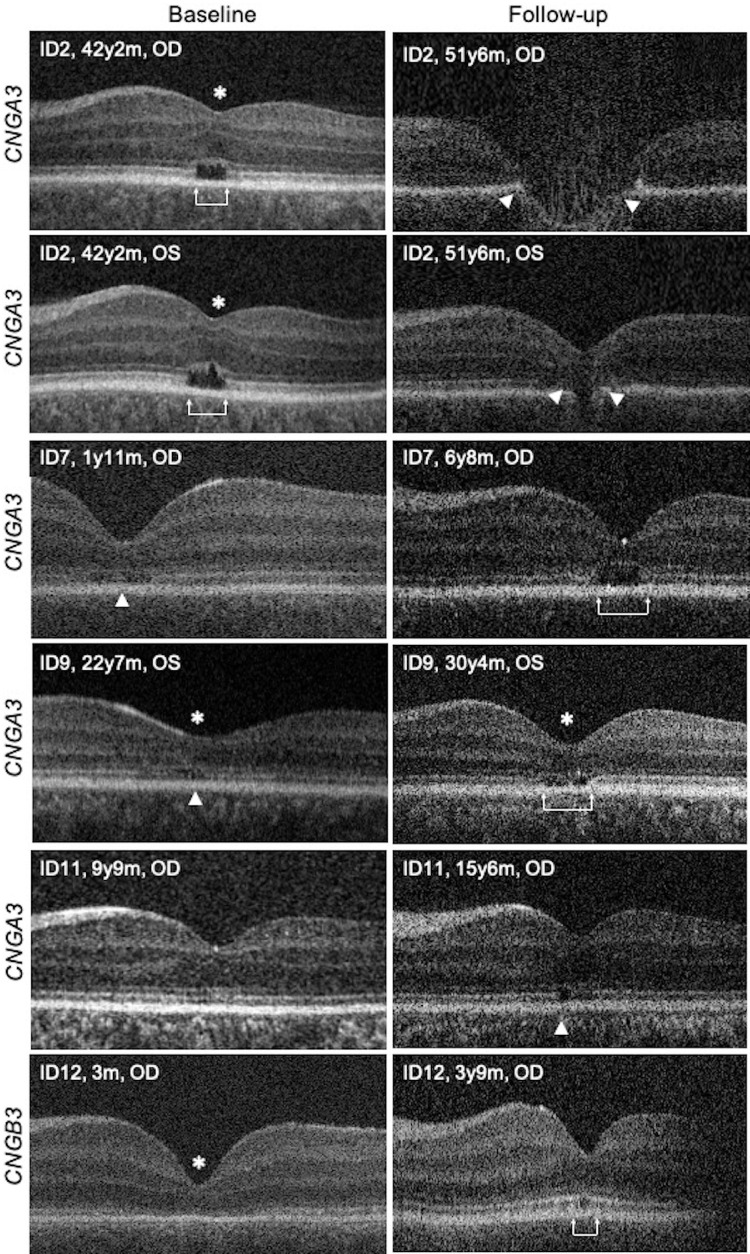
Longitudinal SD-OCT scans of ACHM patients. The *left column* shows horizontal foveal SD-OCT scans at first visit, and the *right column* shows follow-up scans at the same location at the final visit. Representative patterns of progression for all five SD-OCT categories are shown. Foveal hypoplasia is marked with an asterisk. OCT scans from right and left eyes are presented in the same orientation (thickened retinal nerve fiber layer on the left side). Patient ID2 presented with a HRZ at the first visit (*brackets*), and at the final visit she had an RPE/BM break bilaterally (*arrowheads*), with (*OD*) or without (*OS*) retinal excavation. Her refraction was OD −2.50 cylinder (cyl) × 20°, OS −1.50 cyl × 160°. No further predisposing factors have been identified in the patient's medical and ophthalmic history, hence it cannot be determined if the retinal rupture is related to achromatopsia. Patient ID7 presented with EZ absence (*arrowhead*) and progressed to a well-defined HRZ (*brackets*). Patient ID9 presented with EZ disruption (*arrowhead*) and progressed to a HRZ (*brackets*). Patient ID11 had hyperreflective changes, which then progressed to EZ disruption (*arrowhead*). Patient ID12 was three months old and had foveal hypoplasia and EZ absence at first visit. At the age of three months the EZ was probably not fully developed, but became more intact with age. At the final visit after three years, a well-defined HRZ was identified (*brackets*). Hence, the regression of foveal hypoplasia and the increase in ONL thickness may be explained by ongoing foveal development in early childhood.

**Figure 3.**
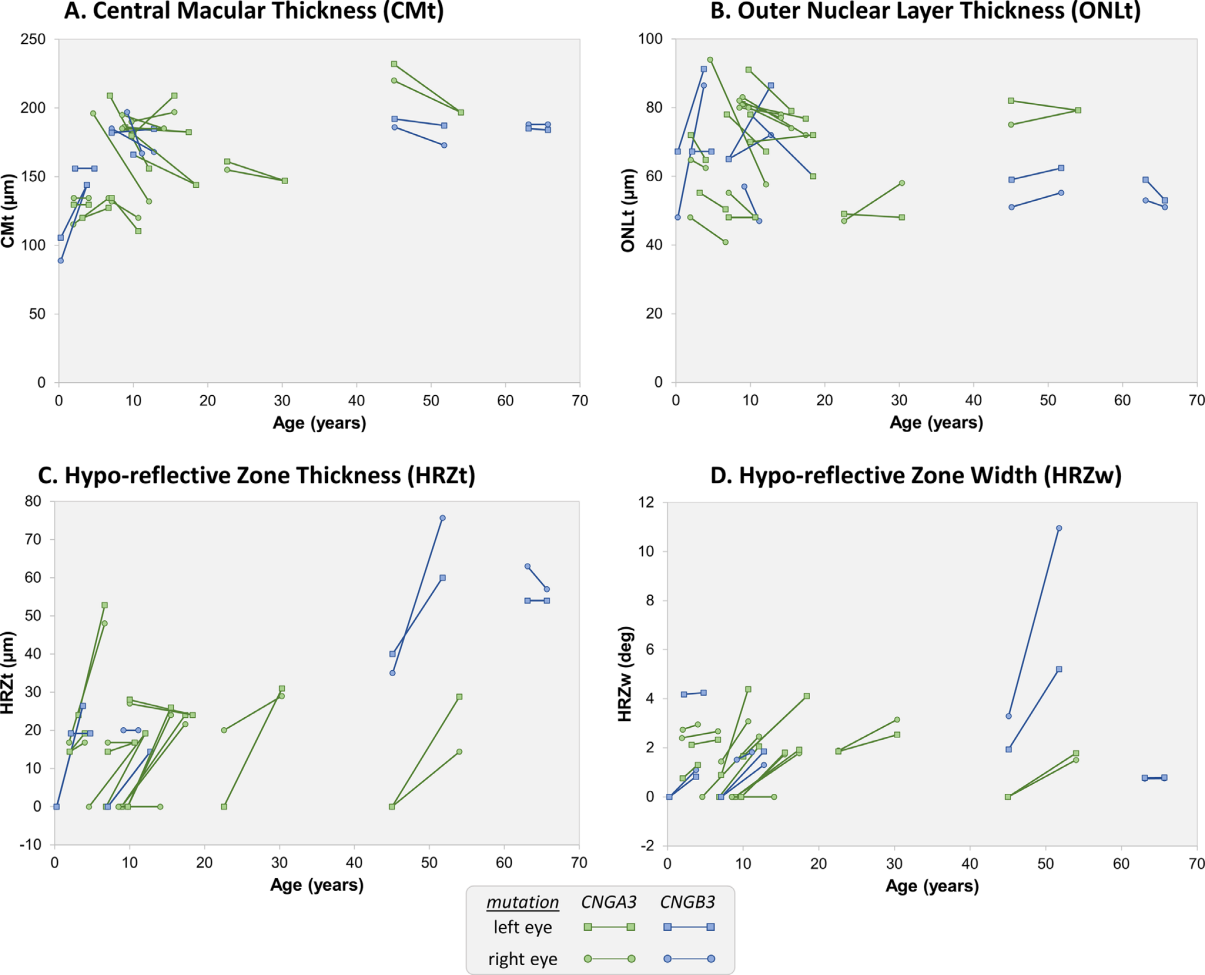
Diagrams showing the changes in CMt (**A**), ONLt (**B**), HRZt (**C**), and HRZw (**D**) between visits. Each line represents values for each eye at first and final visit. (**A**) CMt (µm) increases in patients younger than 10 years old and then decreases in most patients. The change in CMt was nonsignificant (*P* = 0.279). (**B**) ONLt (µm) increases in the youngest patient, decreases in the majority of patients up to 20 years of age, and in the older patients there is some variability in individual changes of ONLt. The change in ONLt was nonsignificant (*P* = 0.812). (**C**) HRZt (µm) increases (*P* = 0.01), indicating that the HRZ gets thicker between visits. (**D**) HRZw (deg) increases (*P* = 0.001), indicating that the HRZ gets wider between visits.

Mean ONLt was 66.8 ± 13.7 µm (range, 47–94 µm) at first visit and 65.5 ± 13.5 µm (range, 40.8–91.2 µm) at the final visit (Fig. 2, Fig. 3B). The change in ONLt between visits was not significant (*P* = 0.812) (mean change per year = −0.24 µm) (Fig. 2). None of these interaction terms were significant for any parameter. The change/year in all the above parameters is presented in Figure 1.

### Hyporeflective Zone

#### Qualitative Analysis

Interexaminer reliability (N = 2) was excellent for qualitative grading of OCT images (intraclass correlation coefficient = 0.92; 95% confidence interval = 0.855, 0.959). Fifteen of 17 patients (88%) showed longitudinal retinal changes for qualitative grading^10^ across a mean follow-up period of 5.7 years (Table). The most common pattern of progression (six patients) was development of an HRZ or “punched out” lesion or category 4.^10^ The remaining patients progressed to EZ disruption (five patients), EZ absence (two patients), and outer retinal atrophy (two patients). Interestingly, two patients (ID1, ID11) presented only with subtle hyperreflectivity changes at the fovea, which subsequently developed to EZ disruption and early hyporeflective changes. Patient ID2 was excluded from quantitative analysis due to development of an RPE/Bruch's membrane (RPE/BM) break on both eyes (Fig. 1, patient ID2). The retinal changes described above are shown in Figure 2 and Supplementary 2.

#### Quantitative Analysis

On quantitative measurement, after including age into the statistical model, there was a significant increase in HRZt (*F* = 7.21; *P* = 0.01) and HRZw (*F* = 13.08; *P* = 0.001) between the two visits, indicating progression of foveal retinal changes between visits. Mean HRZt at the point of maximal diameter was 13.57 ± 17.3 µm at first visit and 27.44 ± 17.4 µm at the final visit (mean increase = [13.87 µm]), with a mean increase rate of 2.51 µm/year (Fig. 2). Mean HRZw was 0.99 ± 1.2° at first visit and 2.48 ± 2.1° at the final visit (mean increase = [1.49°]) with a mean increase rate of 0.26°/y (Fig. 2). The temporal changes of HRZt and HRZw are shown in Figures 3C and 3D. Enlargement of both HRZt and HRZw is present across all age groups included in this study, with some variability observed in the magnitude of individual changes. The fact that changes in HRZt and HRZw are significant between visits, but the interaction term between age and visit is not significant, indicates that disease progression occurs across the entire lifespan. This finding is shown in Figures 3C and 3D where the general trend across all age groups is for the HRZ to get thicker (C) and wider (D).

## Discussion

In this OCT study, we have shown longitudinal retinal changes in 88% of patients with ACHM, with the most common patterns of progression being the development of a HRZ and the transition to EZ disruption. To the best of our knowledge, the present study is one of the longest follow-up studies (mean follow-up: 5.7 years), including a sufficient sample size for statistical analysis. A prior longitudinal study by Hirji et al.^16^ with a mean follow-up of 5.1 years and a recent longitudinal study by Brunetti-Pierri et al.^20^ with a mean follow-up of 5.4 years found that foveal degeneration occurs only in a small subset of patients. In the present study, 15 of 17 patients (88%) demonstrated longitudinal qualitative OCT changes of one (n = 12) or two (n = 3) grades over the follow-up period (Table). This agrees with our longitudinal quantitative assessment of HRZ dimensions (width and thickness), which showed significant changes in the cohort over the time course of this study. Most importantly, enlargement of both HRZt and HRZw was present across all age groups included in this study. This study included participants from a broad age range, ages 0 to 63 years, indicating that disease progression occurs across the lifespan. The retinal changes included development of EZ disruption or HRZ, increase in the size of EZ disruption and HRZ, and development of retinal atrophy. An interesting finding was the foveal hyper-reflectivity changes, which subsequently developed to EZ disruption and early hyporeflective changes in patients (ID1, ID11). Thomas et al. have first described an outer retinal hyperreflective zone in younger patients, which appears to precede the EZ disruption and hyporeflective zone seen in later ages.^13^ It was hypothesized that the increased reflectivity of the foveal cone outer segments may be an early sign of cone photoreceptor degeneration.^13^ Greenberg et al. have also described hyperreflectivity of the external limiting membrane in the fovea and the photoreceptor inner segments.^29^

### Literature Controversy

The implementation of high-resolution imaging of the human retina from a very young age has raised controversy with regards to the progressive nature of ACHM. Thiadens et al.^12^ first suggested that cone cell loss progresses over time and that retinal thickness strongly correlates with age. Thomas et al.^13^^,^^14^ also described age-dependent presence of HRZ and ONL thinning, which was later confirmed in longitudinal studies.^18^ However, Genead et al.^9^ and Sundaram et al.^10^ suggested that cone loss and further OCT parameters, such as foveal retinal thickness and ONL thickness, were not age-dependent. Subsequent longitudinal studies also concluded that the retinal changes in ACHM either remain stable or progress minimally.^15^^,^^16^^,^^19^^,^^20^ The reason for this discrepancy could be explained on the basis of several factors, such as the age at examination, duration of follow-up, use of qualitative versus quantitative measures, and cross-sectional versus longitudinal design. We review each of these factors below in regard to the present findings.

### Age at Examination

Our results are in agreement with the first longitudinal study by Thomas et al.,^13^ who reported that all children with ACHM in their cohort (aged 5–9 years) showed altered foveal cone photoreceptor morphology over an average follow-up of 16 months, while in the older patients there were no gross morphological changes. The consistency with our findings is possibly explained by the similar age distribution of patients, as the majority of patients (12 of 17) in the present study were between two to 10 years old. However, we also found longitudinal retinal changes in four of five adults, indicating disease progression over the entire lifespan. The older age distribution in the study by Aboshiha et al.^15^ (only three patients under 10 years) may be one of the reasons as to why they found qualitative progressive changes in only 5% of their 38 patients. Similarly, the studies by Hirji et al.^16^ and Brunetti-Pierri et al.^20^ included fewer children (seven and six, respectively) than the present study, and this may be one of the reasons that both studies showed progressive retinal changes only in a small number of patients, 12% and 12.5%, respectively. Nevertheless in the study by Brunetti-Pierri et al.^20^ older patients had a lower central retinal thickness and a more advanced OCT grade, suggesting that changes in retinal structure occur slowly, for instance over decades of life. Further cross-sectional studies including many young children with ACHM also found that in early childhood foveal pathology is milder than reported in older patients, suggesting progression with time.^11^^,^^17^ Evidence toward more dynamic retinal changes during the early stages of ACHM is also provided by a longitudinal study by Lee et al.,^18^ who showed that retinal development is ongoing albeit at a reduced rate in children with ACHM (mean age 3.8 years). For example, in patient ID12 foveal hypoplasia was seen (Fig. 1, ID12). However, the patient was three months old at first visit; therefore this finding may be attributed to ongoing foveal development in early childhood.^13^^,^^18^ At final visit after three years, a well-defined HRZ was identified. These findings suggest that gene therapy should be considered at early ages, before there are visible photoreceptor changes on OCT. Recent gene trials have also pointed out that treatment of younger patients may result in greater functional gains by avoiding amblyopia.^30^

### Quantitative Versus Qualitative Measurements

The discrepancies among studies investigating progression of retinal changes in ACHM may be partially attributed to the use of qualitative versus quantitative parameters to assess progression. Our results were based in both qualitative and quantitative analysis of the size of HRZ and EZ disruption, because an a priori qualitative classification may not identify subtle quantitative changes in the retina. We also performed a quantitative assessment considering that the site of EZ disruption can be considered as a small HRZ.

### Duration of Follow-up

In contrast to our findings, previous longitudinal studies of patients with ACHM found progression in OCT grade only in a small proportion of subjects.^15^^,^^16^^,^^19^^,^^20^ Interestingly, retinal changes were noted primarily in younger subjects.^15^^,^^16^^,^^19^^,^^20^ Hence, there may be some progression very early in the disease, which stabilizes later in the first decade.^16^ Additionally, in the study by Langlo et al.,^19^ the follow-up period of 12.8 months was possibly not long enough to detect structural changes, suggesting that alterations in retinal structure may occur over decades of life.^20^ Hence, progressive changes may present in a greater proportion of individuals, if patients are monitored for periods longer than five years.

### CMT and ONL Thickness

Mean final CMt and ONLt in our study were 161.9 µm and 65.5 µm respectively, which are comparable to the values of Sundaram et al.^10^ Most cross-sectional studies generally agree that total retinal thickness and ONL thickness are lower in ACHM patients than in controls.^10^^–^^12^^,^^14^^,^^17^^,^^31^^–^^33^ Regarding the age-dependency of retinal thinning, there are controversial findings. Several cross-sectional studies suggested an age-dependent development of HRZ and ONL thinning, because older patients demonstrated more commonly a HRZ and severe foveal atrophy.^9^^,^^14^^,^^17^ By contrast, other authors did not find significant longitudinal ONL or CMT thinning.^16^ This study showed minimal decreases in CMt and ONLt, which were not significant. This is probably related to the inclusion of very young children, whereby the retina is still developing. In particular, CMt tended to increase in patients younger than 10 years old, and then decrease in most patients (Fig. 1, patient ID12, Fig. 3A). By including an interaction term between age and visit in the statistical model, our findings suggest that CMt tends to increase with age and decrease with disease progression; however, these results did not reach significance. In agreement with this, Figure 3B shows that ONLt increases in the youngest patient, although it decreases thereafter and shows minimal variation in the older subjects. Previous studies indeed suggested that in the first years of life ONLt of ACHM patients increases with age, although the ONL is thinner in ACHM than in controls.^18^ Studies including many children under 10 years have found small but significant increases in ONLt; hence, the increase in ONLt might be related to ongoing retinal development in younger subjects.^16^^,^^19^ Additionally, in the study by Thomas et al.,^13^ patients under 10 years of age showed larger reductions in CMt and ONLt than patients older than 40 years, who had minimal changes in CMt and ONLt. On the other hand, studies including few children under 10 years old found no significant change in ONLt between visits.^10^^,^^15^^,^^32^ The fact that ONLt does not show the same pattern of change for all age groups further supports the idea that therapeutic interventions in ACHM should be implemented at an earlier age.^13^^,^^17^^,^^18^

## Conclusion

In conclusion, our study, which includes a large number of children and a long follow-up, showed that most patients (88%) have longitudinal outer retinal changes, and a significant increase of the HRZ. Additionally, progression of retinal changes was evident across the entire lifespan. Limitations of this study are the lack of genotypic homogeneity (11 patients with CNGA3 mutation and six patients with CNGB3 mutation) and the phenotypic variability in the degree of progression, even within individuals with the same genotype. Although progressive retinal changes were demonstrated in 88% of patients, these results cannot be generalized to all patients with ACHM. Additionally, our findings in adults must be interpreted with caution because of the small sample size. The pediatric group might also benefit from a larger sample and possibly a stratification by age, also in consideration of the ongoing foveal development in younger ACHM subjects. To summarize, the progression of foveal changes strongly advocates early treatment for ACHM, before the development of visible photoreceptor changes on OCT. Altered organization of the visual cortex has been considered as potential limiting factor for efficacy in adult patients.^34^ Hence, future gene therapies should investigate if pediatric patients are better candidates than adults, due to higher cortical plasticity.^21^^,^^34^ Structural OCT parameters, such as the dimensions of the HRZ, could be used as surrogate endpoints, as they are probably more sensitive and precede the deterioration of functional measures, such as the BCVA. Inter-patient variability in structural foveal parameters may also affect the therapeutic potential of gene treatments, and previous adaptive optics scanning light ophthalmoscopy (AOSLO) studies have shown that the number and spatial distribution of foveal cones are highly variable across patients with CNGB3-associated ACHM.^11^^,^^35^ Although we did not find differences between patients with ACHM due to *CNGA3* or *CNGB3* mutations, large studies including patients with all genetic mutations, as well as very young children, are warranted.

## Supplementary Material

Supplement 1

Supplement 2
