# Supplementary material for: Longitudinal Evaluation of Changes in Retinal Architecture Using Optical Coherence Tomography in Achromatopsia
Source: Invest Ophthalmol Vis Sci. 2022 Aug 5;63(9):6. doi: 10.1167/iovs.63.9.6 (PMC9363676; doi:10.1167/iovs.63.9.6)
Supplement: Supplement 1 [file iovs-63-9-6_s001.pdf]

Acquisition protocols for the OCT devices used in this study

OCT Copernicus
For the SOCT Copernicus, a 3-dimensional scan program (743x75; A scans x B scans) was used to capture foveal and parafoveal regions. The scanning window covered a 7x7-mm retinal area, centered at the fovea. The effective axial and transverse resolutions obtained using this machine were approximately 3 and 12 μm, respectively, with a scanning speed of 52,000 A-scans/second.

Hand-held OCT
HH SD-OCT (ENVISU C class 2300; scan depth, 3.4 mm; 32.000 A-scans per second) uses a broadband infrared light source with a wavelength of 870 nm, yielding a theoretic axial resolution of 3.6 μm in air and 2.6 μm in tissue. A 3-dimensional raster scan program consisting of 100 B-scans and 500 A-scans per B-scan line was used to capture the foveal and parafoveal regions. The scanning window covered a 10x5-mm retinal area centered on the fovea.
