# Supplementary material for: Longitudinal Evaluation of Changes in Retinal Architecture Using Optical Coherence Tomography in Achromatopsia
Source: Invest Ophthalmol Vis Sci. 2022 Aug 5;63(9):6. doi: 10.1167/iovs.63.9.6 (PMC9363676; doi:10.1167/iovs.63.9.6)
Supplement: Supplement 2 [file iovs-63-9-6_s002.pdf]

ID	Visit 1					Visit 2				
	Age	OD		OS		Age	OD		OS	
**1**	45y	CNGA3<br>HRZt 0<br>HRZw 0<br>ONLt 75	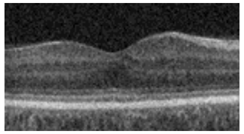	CNGA3<br>HRZt 0<br>HRZw 0<br>ONLt 82	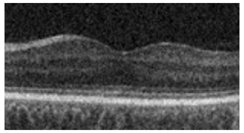	54y	*CNGA3*<br>HRZt 14.4<br>HRZw 1.50<br>ONLt 79.2	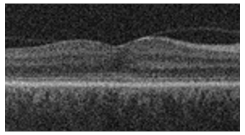	CNGA3<br>HRZt 28.8<br>HRZw 1.78<br>ONLt 79.2	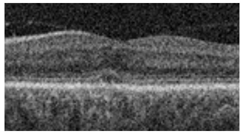
**2**	42y<br>2m	CNGA3<br>Excluded	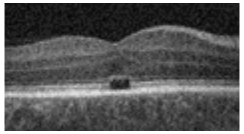	Missing<br>value	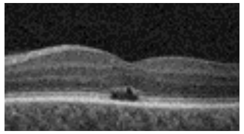	51y<br>6m	Missing<br>value	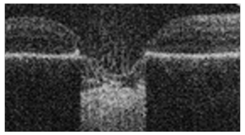	Missing<br>value	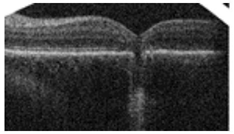
**3**	8y<br>11m	CNGA3<br>HRZt 0<br>HRZw 0<br>ONLt 83	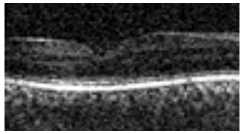	CNGA3<br>HRZt 0<br>HRZw 0<br>ONLt 81	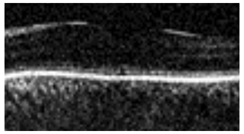	17y<br>5m	CNGA3<br>HRZt 21.6<br>HRZw 1.78<br>ONLt 72	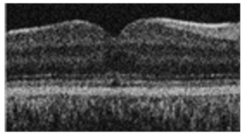	CNGA3<br>HRZt 24<br>HRZw 1.92<br>ONLt 76.8	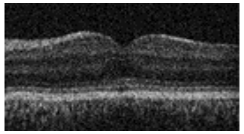
**4**	63y<br>1m	CNGB3<br>HRZt 63<br>HRZw 0.74<br>ONLt 53	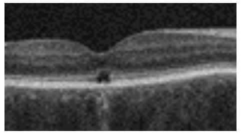	CNGB3<br>HRZt 54<br>HRZw 0.77<br>ONLt 59	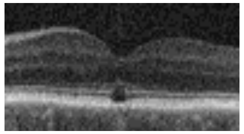	65y<br>8m	CNGB3<br>HRZt 57<br>HRZw 0.75<br>ONLt 51	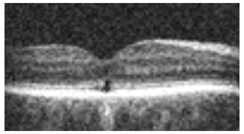	CNGB3<br>HRZt 54<br>HRZw 0.78<br>ONLt 53	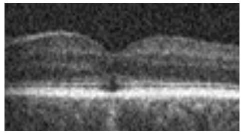
**5**	4y<br>7m	CNGA3<br>HRZt 0<br>HRZw 0<br>ONLt 94	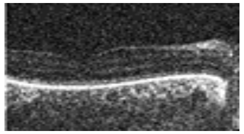	CNGA3<br>HRZt 0<br>HRZw 0<br>ONLt 78	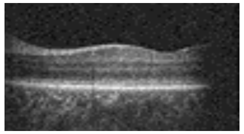	12y<br>1m	CNGA3<br>HRZt 19.2<br>HRZw 2.47<br>ONLt 57.6	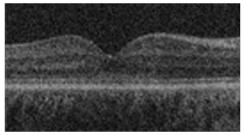	CNGA3<br>HRZt 19.2<br>HRZw 2.05<br>ONLt 67.2	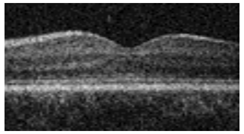
**6**	7y<br>1m	CNGB3<br>HRZt 0<br>HRZw 0<br>ONLt 65	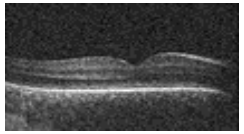	CNGB3<br>HRZt 0<br>HRZw 0<br>ONLt 65	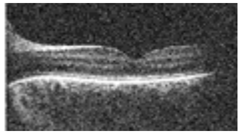	12y<br>9m	CNGB3<br>HRZt 14.4<br>HRZw 1.30<br>ONLt 72	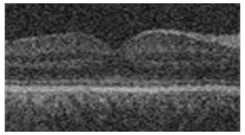	CNGB3<br>HRZt 14.4<br>HRZw 1.85<br>ONLt 86.4	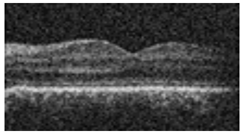
**7**	1y<br>11m	CNGA3<br>HRZt 16.8<br>HRZw 2.40<br>ONLt 48	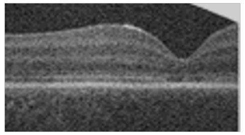	CNGA3<br>HRZt 24<br>HRZw 2.12<br>ONLt 55.2	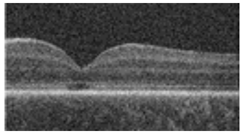	6y<br>8m	CNGA3<br>HRZt 48<br>HRZw 2.67<br>ONLt 40.8	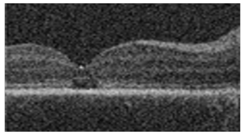	CNGA3<br>HRZt 52.8<br>HRZw 2.33<br>ONLt 50.4	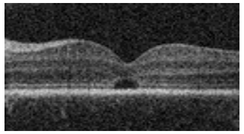
**8**	8y<br>6m	CNGA3<br>HRZt 0<br>HRZw 0<br>ONLt 80	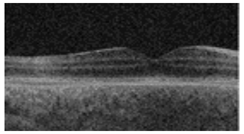	CNGA3<br>HRZt 0<br>HRZw 0<br>ONLt 82	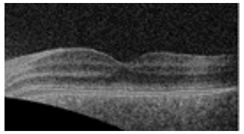	14y<br>1m	CNGA3<br>HRZt 0<br>HRZw 0<br>ONLt 78	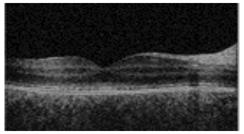	CNGA3<br>HRZt 0<br>HRZw 0<br>ONLt 77	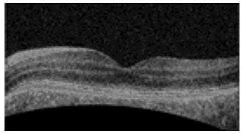
**9**	22y<br>7m	CNGA3<br>HRZt 20<br>HRZw 1.88<br>ONLt 47	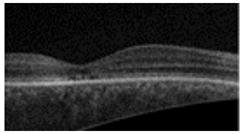	CNGA3<br>HRZt 0<br>HRZw 1.84<br>ONLt 49	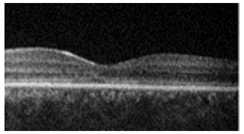	30y<br>4m	CNGA3<br>HRZt 29<br>HRZw 3.15<br>ONLt 58	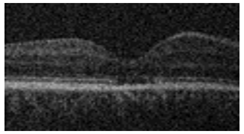	CNGA3<br>HRZt 31<br>HRZw 2.53<br>ONLt 48	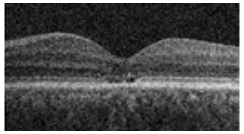

**10**	10y	CNGA3<br>HRZt 27<br>HRZw 1.66<br>ONLt 78	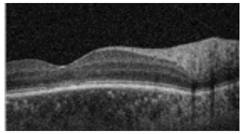	CNGA3<br>HRZt 28<br>HRZw 1.64<br>ONLt 70	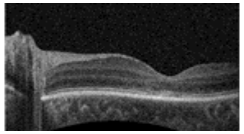	18y<br>5m	CNGA3<br>HRZt 24<br>HRZw 4.11<br>ONLt 60	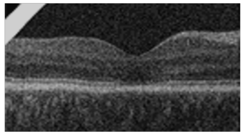	CNGA3<br>HRZt 24<br>HRZw 4.11<br>ONLt 72	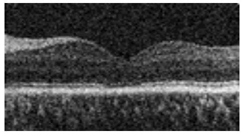
**11**	9y<br>9m	CNGA3<br>HRZt 0<br>HRZw 0<br>ONLt 80	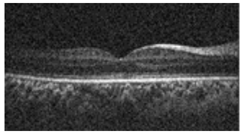	CNGA3<br>HRZt 0<br>HRZw 0<br>ONLt 91	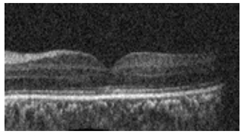	15y<br>6m	CNGA3<br>HRZt 24<br>HRZw 1.74<br>ONLt 74	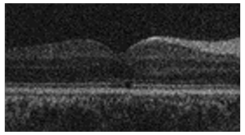	CNGA3<br>HRZt 26<br>HRZw 1.80<br>ONLt 79	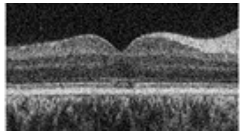
**12**	3m	CNGB3<br>HRZt 0<br>HRZw 0<br>ONLt 48	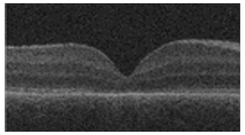	CNGB3<br>HRZt 0<br>HRZw 0<br>ONLt 67.2	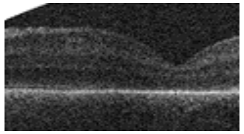	3y<br>9m	CNGB3<br>HRZt 26.4<br>HRZw 1.09<br>ONLt 86.4	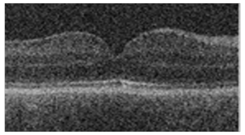	CNGB3<br>HRZt 26.4<br>HRZw 0.82<br>ONLt 91.2	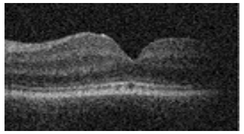
**13**	45y<br>1m	CNGB3<br>HRZt 35<br>HRZw 3.29<br>ONLt 51	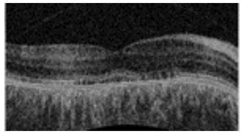	CNGB3<br>HRZt 40<br>HRZw 1.93<br>ONLt 59	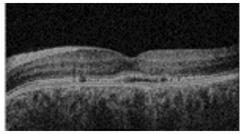	51y<br>9m	CNGB3<br>HRZt 75.7<br>HRZw 10.9<br>ONLt 55.2	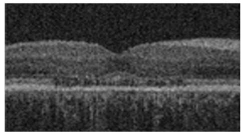	CNGB3<br>HRZt 60<br>HRZw 5.21<br>ONLt 62.4	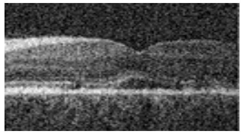
**14**	2y	CNGA3<br>HRZt 14.4<br>HRZw 2.74<br>ONLt 64.8	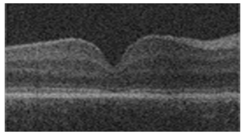	CNGA3<br>HRZt 14.4<br>HRZw 0.75<br>ONLt 72	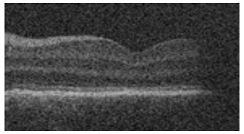	4y	CNGA3<br>HRZt 16.8<br>HRZw 2.95<br>ONLt 62.4	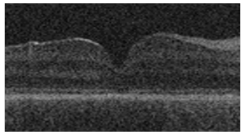	CNGA3<br>HRZt 19.2<br>HRZw 1.30<br>ONLt 64.8	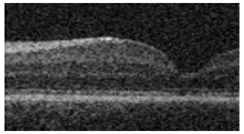
**15**	2y<br>2m	Missing value	Missing value	CNGB3<br>HRZt 19.2<br>HRZw 4.18<br>ONLt 67.2	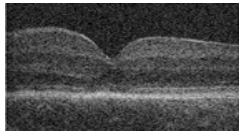	4y<br>9m	Missing value	Missing value	CNGB3<br>HRZt 19.2<br>HRZw 4.25<br>ONLt 67.2	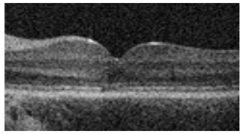
**16**	9y<br>2m	CNGB3<br>HRZt 20<br>HRZw 1.52<br>ONLt 57	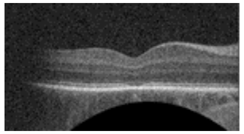	Missing value	Missing value	11y<br>2m	CNGB3<br>HRZt 20<br>HRZw 1.82<br>ONLt 47	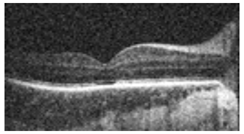	Missing value	Missing value
**17**	7y<br>1m	CNGA3<br>HRZt 16.8<br>HRZw 1.44<br>ONLt 55.2	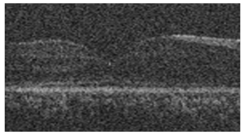	CNGA3<br>HRZt 14.4<br>HRZw 0.89<br>ONLt 48	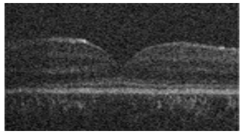	10y<br>9m	CNGA3<br>HRZt 16.8<br>HRZw 3.08<br>ONLt 48	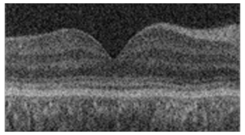	CNGA3<br>HRZt 16.8<br>HRZw 4.38<br>ONLt 48	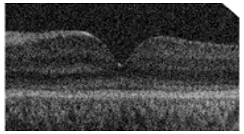

Supplement 2. Summary of study participants and corresponding OCT scans. y:years, m:months, HRZ: hyporeflective zone, HRZt: vertical hyporeflective zone thickness in µm, HRZw: horizontal hyporeflective zone thickness in degrees, ONLt: outer nuclear layer thickness in µm
